# Supplementary material for: Enrichment of rare codons at 5' ends of genes is a spandrel caused by evolutionary sequence turnover and does not improve translation
Source: eLife. 2024 Jul 15;12:RP89656. doi: 10.7554/eLife.89656 (PMC11249729; doi:10.7554/eLife.89656)
Supplement: Supplementary file 3. — Scores were calculated as described in Materials and methods, but in this example, only for a subset of Saccharomycotina. ‘Total Hits’ is the number of different proteins from the sub-phylum Saccharomycotina subset giving a BLAST bit-score of at least 50. ‘Hits in the first 40 amino acids’ is the number of proteins (out of the proteins in the ‘Total Hits’ columns) that had a BLAST alignment with an alignment score >200 matching any part of the first 40 amino acids of the query sequence (i.e. of PCA1, NSR1, etc.). ‘Query Start’ is the range of amino acid positions in the Query protein where the BLAST alignments started. For instance, for BUD5, the 125 Saccharomycotina homologs had BLAST alignments that started at positions between amino acid 211 and amino acid 420 on S. cerevisiae BUD5; none had an alignment starting within the first 40 amino acids. For SNX41, 65 of the 121 hits had an alignment beginning within the first 40 amino acids of S. cerevisiae SNX41. For RPL12B, all 121 of the Saccharomycotina homologs had BLAST alignments starting at amino acid 1 of S. cerevisiae RPL12B. The ‘Conservation Score’ is the score calculated as described in Materials and methods. Note that the number of hits varies in part because the genomes of the Saccharomycotina species were not all fully sequenced. Thus, BNA2 likely has fifteen fewer hits than TRP3 because the BNA2 locus was not sequenced in some species. However, the number of hits does not affect the conservation score, as long as the number meets the qualifying minimum. [file elife-89656-supp3.docx]

Supplementary File 3. Example conservation scores.

| **Protein** | **Total Hits** | **Hits in the first 40 amino acids** | **Query Start** | **Conservation Score** |
| --- | --- | --- | --- | --- |
| BUD5 | 125 | 0 | 211-420 | 0 |
| NSR1 | 122 | 0 | 61-264 | 0 |
| SNX41 | 121 | 65 | 2-199 | 10.7 |
| BNA2 | 107 | 106 | 15-86 | 20.8 |
| TRP3 | 122 | 122 | 1-23 | 30.6 |
| RPL12B | 121 | 121 | 1 | 40 |
