# Supplementary material for: Enrichment of rare codons at 5' ends of genes is a spandrel caused by evolutionary sequence turnover and does not improve translation
Source: eLife. 2024 Jul 15;12:RP89656. doi: 10.7554/eLife.89656 (PMC11249729; doi:10.7554/eLife.89656)
Supplement: Supplementary file 4. — ‘Query start position in BLAST alignment’ is the amino acid residue of the S. cerevisiae query protein where a BLAST alignment (alignment score >200) begins with a protein of Saccharomycotina. ‘Proportion of hits with this Q-Start Position’ is the proportion of qualifying Saccharomycotina hits (i.e. bit-score >50) that have their alignment begin at this position. ‘Weight’ is multiplied by ‘Proportion,’ and the sum is the conservation score. [file elife-89656-supp4.docx]

Supplemental File 4. Calculation of a conservation score.

| Query start position in BLAST alignment | Weight to give | Proportion of hits with this Q-Start Position | Weight x Proportion |
| --- | --- | --- | --- |
| 1 | 40 | 0.22 | 8.8 |
| 2 | 39 | 0.01 | 0.39 |
| 3 | 38 | 0.01 | 0.38 |
| 4 | 37 | 0.02 | 0.74 |
| . | . | . | . |
| . | . | . | . |
| . | . | . | . |
| 38 | 3 | 0.03 | 0.09 |
| 39 | 2 | 0.03 | 0.06 |
| 40 | 1 | 0.04 | 0.04 |
|  |  |  |  |
|  |  |  | Sum (= Cons. Score) |
