# Supplementary material for: Enrichment of rare codons at 5' ends of genes is a spandrel caused by evolutionary sequence turnover and does not improve translation
Source: eLife. 2024 Jul 15;12:RP89656. doi: 10.7554/eLife.89656 (PMC11249729; doi:10.7554/eLife.89656)
Supplement: Supplementary file 5. [file elife-89656-supp5.docx]

Supplementary File 5. Sequences of Ramp genes in Figure 8.

Ramp 1. MIT

ATGACAGAGCAAAACGCCCTAGTAAAGCGTATTACAAATCAAACCAAGATTCAGATTGCGATCTCTTTAAAGGGTGGTCCCCTAGCGATAGAGCACTCGATCTTCCCAGAAAAAGAGGCAAGTACAGAGGTCACTTTCGATACTGCCTCTACTTCTGGGTTAGAGGCGCTGTTTCAGGGACCTGGCCCTGGCCGTATATTC*AATGAT*TACCCCTACGACGTACCTGACTACGCTGGCTCCGCTGCAGTTATCTCCAAGGGTGAGGAGTTGTTTACAGGGGTCGTACCGATCTTGGTCGAGTTGGACGGGGACGTCAACGGACATAAGTTCAGCGTGAGAGGCGAAGGAGAGGGAGACGCAACGAATGGGAAGCTGACACTAAAGTTCATCTGTACAACCGGTAAGTTACCAGTCCCTTGGCCTACTTTAGTGACCACATTAACATACGGAGTGCAGTGTTTCTCAAGATACCCTGACCATATGAAGAGACACGATTTCTTTAAATCTGCTATGCCGGAAGGCTACGTTCAAGAGAGGACCATTTCATTTAAGGACGATGGAACATATAAAACCAGAGCAGAGGTGAAATTCGAGGGAGACACACTAGTCAACAGGATTGAACTGAAGGGCATCGACTTCAAGGAAGACGGTAATATATTGGGCCATAAGTTGGAATACAATTTCAATTCTCACAACGTTTATATCACTGCGGATAAGCAGAAGAACGGCATTAAGGCCAATTTTAAGATCCGTCATAATGTCGAGGACGGTTCTGTGCAGTTGGCTGATCACTACCAGCAGAACACACCAATCGGAGACGGTCCTGTATTACTTCCTGATAATCACTATTTATCCACTCAGTCAGTATTATCTAAGGACCCGAATGAGAAAAGAGATCATATGGTGTTGCTAGAATTCGTCACCGCAGCAGGTATAACTCACGGAATGGACGAATTATATAAGGGCAGCTGA

Ramp 2. SIT

ATGACAGAGCAAAACGCGCTAGTAAAGCGGATTACAAATCAGACGAAGATCCAGATCGCGATCAGCCTCAAAGGCGGGCCGCTTGCGATAGAGCACTCGATCTTCCCGGAGAAAGAGGCATCGACAGAGGTCACTTTCGATACTGCCTCTACTTCTGGGTTAGAGGCGCTGTTTCAGGGACCTGGCCCTGGCCGTATATTC*AATGAT*TACCCCTACGACGTACCTGACTACGCTGGCTCCGCTGCAGTTATCTCCAAGGGTGAGGAGTTGTTTACAGGGGTCGTACCGATCTTGGTCGAGTTGGACGGGGACGTCAACGGACATAAGTTCAGCGTGAGAGGCGAAGGAGAGGGAGACGCAACGAATGGGAAGCTGACACTAAAGTTCATCTGTACAACCGGTAAGTTACCAGTCCCTTGGCCTACTTTAGTGACCACATTAACATACGGAGTGCAGTGTTTCTCAAGATACCCTGACCATATGAAGAGACACGATTTCTTTAAATCTGCTATGCCGGAAGGCTACGTTCAAGAGAGGACCATTTCATTTAAGGACGATGGAACATATAAAACCAGAGCAGAGGTGAAATTCGAGGGAGACACACTAGTCAACAGGATTGAACTGAAGGGCATCGACTTCAAGGAAGACGGTAATATATTGGGCCATAAGTTGGAATACAATTTCAATTCTCACAACGTTTATATCACTGCGGATAAGCAGAAGAACGGCATTAAGGCCAATTTTAAGATCCGTCATAATGTCGAGGACGGTTCTGTGCAGTTGGCTGATCACTACCAGCAGAACACACCAATCGGAGACGGTCCTGTATTACTTCCTGATAATCACTATTTATCCACTCAGTCAGTATTATCTAAGGACCCGAATGAGAAAAGAGATCATATGGTGTTGCTAGAATTCGTCACCGCAGCAGGTATAACTCACGGAATGGACGAATTATATAAGGGCAGCTGA

Ramp 3. FIT

ATGACAGAACAAAACGCTTTGGTTAAAAGAATTACAAATCAAACTAAGATTCAAATTGCTATTTCTTTGAAAGGTGGTCCATTAGCTATTGAACATTCTATTTTCCCAGAAAAAGAGGCAAGTACAGAGGTCACTTTCGATACTGCCTCTACTTCTGGGTTAGAGGCGCTGTTTCAGGGACCTGGCCCTGGCCGTATATTC*AATGAT*TACCCCTACGACGTACCTGACTACGCTGGCTCCGCTGCAGTTATCTCCAAGGGTGAGGAGTTGTTTACAGGGGTCGTACCGATCTTGGTCGAGTTGGACGGGGACGTCAACGGACATAAGTTCAGCGTGAGAGGCGAAGGAGAGGGAGACGCAACGAATGGGAAGCTGACACTAAAGTTCATCTGTACAACCGGTAAGTTACCAGTCCCTTGGCCTACTTTAGTGACCACATTAACATACGGAGTGCAGTGTTTCTCAAGATACCCTGACCATATGAAGAGACACGATTTCTTTAAATCTGCTATGCCGGAAGGCTACGTTCAAGAGAGGACCATTTCATTTAAGGACGATGGAACATATAAAACCAGAGCAGAGGTGAAATTCGAGGGAGACACACTAGTCAACAGGATTGAACTGAAGGGCATCGACTTCAAGGAAGACGGTAATATATTGGGCCATAAGTTGGAATACAATTTCAATTCTCACAACGTTTATATCACTGCGGATAAGCAGAAGAACGGCATTAAGGCCAATTTTAAGATCCGTCATAATGTCGAGGACGGTTCTGTGCAGTTGGCTGATCACTACCAGCAGAACACACCAATCGGAGACGGTCCTGTATTACTTCCTGATAATCACTATTTATCCACTCAGTCAGTATTATCTAAGGACCCGAATGAGAAAAGAGATCATATGGTGTTGCTAGAATTCGTCACCGCAGCAGGTATAACTCACGGAATGGACGAATTATATAAGGGCAGCTGA

Ramp 4 MIT with PCS

ATGACAGAGCAAAACGCCCTAGTAAAGCGTATTACAAATCAAACCAAGATTCAGATTGCGATCTCTTTAAAGGGTGGTCCCCTAGCGATAGAGCACTCGATCTTCCCAGAAAAAGAGGCAAGTACAGAGGTCACTTTCGATACTGCCTCTACTTCTGGGTTAGAGGCGCTGTTTCAGGGACCTGGCCCTGGCCGTATATTC*CGACGG*TACCCCTACGACGTACCTGACTACGCTGGCTCCGCTGCAGTTATCTCCAAGGGTGAGGAGTTGTTTACAGGGGTCGTACCGATCTTGGTCGAGTTGGACGGGGACGTCAACGGACATAAGTTCAGCGTGAGAGGCGAAGGAGAGGGAGACGCAACGAATGGGAAGCTGACACTAAAGTTCATCTGTACAACCGGTAAGTTACCAGTCCCTTGGCCTACTTTAGTGACCACATTAACATACGGAGTGCAGTGTTTCTCAAGATACCCTGACCATATGAAGAGACACGATTTCTTTAAATCTGCTATGCCGGAAGGCTACGTTCAAGAGAGGACCATTTCATTTAAGGACGATGGAACATATAAAACCAGAGCAGAGGTGAAATTCGAGGGAGACACACTAGTCAACAGGATTGAACTGAAGGGCATCGACTTCAAGGAAGACGGTAATATATTGGGCCATAAGTTGGAATACAATTTCAATTCTCACAACGTTTATATCACTGCGGATAAGCAGAAGAACGGCATTAAGGCCAATTTTAAGATCCGTCATAATGTCGAGGACGGTTCTGTGCAGTTGGCTGATCACTACCAGCAGAACACACCAATCGGAGACGGTCCTGTATTACTTCCTGATAATCACTATTTATCCACTCAGTCAGTATTATCTAAGGACCCGAATGAGAAAAGAGATCATATGGTGTTGCTAGAATTCGTCACCGCAGCAGGTATAACTCACGGAATGGACGAATTATATAAGGGCAGCTGA

Ramp 5 SIT with PCS.

ATGACAGAGCAAAACGCGCTAGTAAAGCGGATTACAAATCAGACGAAGATCCAGATCGCGATCAGCCTCAAAGGCGGGCCGCTTGCGATAGAGCACTCGATCTTCCCGGAGAAAGAGGCATCGACAGAGGTCACTTTCGATACTGCCTCTACTTCTGGGTTAGAGGCGCTGTTTCAGGGACCTGGCCCTGGCCGTATATTC*CGACGG*TACCCCTACGACGTACCTGACTACGCTGGCTCCGCTGCAGTTATCTCCAAGGGTGAGGAGTTGTTTACAGGGGTCGTACCGATCTTGGTCGAGTTGGACGGGGACGTCAACGGACATAAGTTCAGCGTGAGAGGCGAAGGAGAGGGAGACGCAACGAATGGGAAGCTGACACTAAAGTTCATCTGTACAACCGGTAAGTTACCAGTCCCTTGGCCTACTTTAGTGACCACATTAACATACGGAGTGCAGTGTTTCTCAAGATACCCTGACCATATGAAGAGACACGATTTCTTTAAATCTGCTATGCCGGAAGGCTACGTTCAAGAGAGGACCATTTCATTTAAGGACGATGGAACATATAAAACCAGAGCAGAGGTGAAATTCGAGGGAGACACACTAGTCAACAGGATTGAACTGAAGGGCATCGACTTCAAGGAAGACGGTAATATATTGGGCCATAAGTTGGAATACAATTTCAATTCTCACAACGTTTATATCACTGCGGATAAGCAGAAGAACGGCATTAAGGCCAATTTTAAGATCCGTCATAATGTCGAGGACGGTTCTGTGCAGTTGGCTGATCACTACCAGCAGAACACACCAATCGGAGACGGTCCTGTATTACTTCCTGATAATCACTATTTATCCACTCAGTCAGTATTATCTAAGGACCCGAATGAGAAAAGAGATCATATGGTGTTGCTAGAATTCGTCACCGCAGCAGGTATAACTCACGGAATGGACGAATTATATAAGGGCAGCTGA

Ramp 6 FIT with PCS.

ATGACAGAACAAAACGCTTTGGTTAAAAGAATTACAAATCAAACTAAGATTCAAATTGCTATTTCTTTGAAAGGTGGTCCATTAGCTATTGAACATTCTATTTTCCCAGAAAAAGAGGCAAGTACAGAGGTCACTTTCGATACTGCCTCTACTTCTGGGTTAGAGGCGCTGTTTCAGGGACCTGGCCCTGGCCGTATATTC*CGACGG*TACCCCTACGACGTACCTGACTACGCTGGCTCCGCTGCAGTTATCTCCAAGGGTGAGGAGTTGTTTACAGGGGTCGTACCGATCTTGGTCGAGTTGGACGGGGACGTCAACGGACATAAGTTCAGCGTGAGAGGCGAAGGAGAGGGAGACGCAACGAATGGGAAGCTGACACTAAAGTTCATCTGTACAACCGGTAAGTTACCAGTCCCTTGGCCTACTTTAGTGACCACATTAACATACGGAGTGCAGTGTTTCTCAAGATACCCTGACCATATGAAGAGACACGATTTCTTTAAATCTGCTATGCCGGAAGGCTACGTTCAAGAGAGGACCATTTCATTTAAGGACGATGGAACATATAAAACCAGAGCAGAGGTGAAATTCGAGGGAGACACACTAGTCAACAGGATTGAACTGAAGGGCATCGACTTCAAGGAAGACGGTAATATATTGGGCCATAAGTTGGAATACAATTTCAATTCTCACAACGTTTATATCACTGCGGATAAGCAGAAGAACGGCATTAAGGCCAATTTTAAGATCCGTCATAATGTCGAGGACGGTTCTGTGCAGTTGGCTGATCACTACCAGCAGAACACACCAATCGGAGACGGTCCTGTATTACTTCCTGATAATCACTATTTATCCACTCAGTCAGTATTATCTAAGGACCCGAATGAGAAAAGAGATCATATGGTGTTGCTAGAATTCGTCACCGCAGCAGGTATAACTCACGGAATGGACGAATTATATAAGGGCAGCTGA
